# Metabolomics analysis of milk thistle lipids to identify drought-tolerant genes

**DOI:** 10.1038/s41598-022-16887-9

**Published:** 2022-07-27

**Authors:** Rahele Ghanbari Moheb Seraj, Masoud Tohidfar, Maryam Azimzadeh Irani, Keyvan Esmaeilzadeh-Salestani, Toktam Moradian, Asadollah Ahmadikhah, Mahdi Behnamian

**Affiliations:** 1 Department of Horticultural Sciences, Faculty of Agriculture and Natural Resources, University of Mohaghegh Ardabili, Ardabil, Iran; 2 Department of Plant Biotechnology, Faculty of Life Sciences and Biotechnology, Shahid Beheshti University, Tehran, Iran; 3 Faculty of Life Sciences and Biotechnology, Shahid Beheshti University, Tehran, Iran; 4 Chair of Crop Science and Plant Biology, Institute of Agricultural and Environmental Sciences, Estonian University of Life Sciences, Fr. R. Kreutzwaldi 1, 51014 Tartu, Estonia; 5 Department of Horticultural Sciences, Islamic Azad University, Shirvan Branch, Shirvan, Iran

**Keywords:** Biotechnology, Computational biology and bioinformatics, Plant sciences

## Abstract

Milk thistle is an oil and medicinal crop known as an alternative oil crop with a high level of unsaturated fatty acids, which makes it a favorable edible oil for use in food production. To evaluate the importance of Milk thistle lipids in drought tolerance, an experiment was performed in field conditions under three different water deficit levels (Field capacity (FC), 70% FC and 40% FC). After harvesting seeds of the plant, their oily and methanolic extracts were isolated, and subsequently, types and amounts of lipids were measured using GC–MS. Genes and enzymes engaged in biosynthesizing of these lipids were identified and their expression in Arabidopsis was investigated under similar conditions. The results showed that content of almost all measured lipids of milk thistle decreased under severe drought stress, but genes (belonged to Arabidopsis), which were involved in their biosynthetic pathway showed different expression patterns. Genes biosynthesizing lipids, which had significant amounts were selected and their gene and metabolic network were established. Two networks were correlated, and for each pathway, their lipids and respective biosynthesizing genes were grouped together. Four up-regulated genes including *PXG3*, *LOX2*, *CYP710A1*, *PAL* and 4 down-regulated genes including *FATA2*, *CYP86A1*, *LACS3*, *PLA2-ALPHA* were selected. The expression of these eight genes in milk thistle was similar to Arabidopsis under drought stress. Thus, *PXG3*, *PAL*, *LOX2* and *CYP86A1* genes that increased expression were selected for protein analysis. Due to the lack of protein structure of these genes in the milk thistle, modeling homology was performed for them. The results of molecular docking showed that the four proteins CYP86A1, LOX2, PAL and PXG3 bind to ligands HEM, 11O, ACT and LIG, respectively. HEM ligand was involved in production of secondary metabolites and dehydration tolerance, and HEM binding site remained conserved in various plants. CA ligands were involved in synthesis of cuticles and waxes. Overall, this study confirmed the importance of lipids in drought stress tolerance in milk thistle.

## Introduction

Metabolomics, as a comprehensive metabolic profiling approach, enables a wide range of metabolite classes be analyzed simultaneously using bioinformatics method^1^. Recently, data acquired from genomics and metabolomics studies as well as application of tools to characterize biosynthetic pathways of enzymes have provided the understanding of processes engaged in production of plant metabolites^2^. Study of compounds derived from medicinal and aromatic plants, which have been used to make approximately 25% of drugs in use by humanity, improves our knowledge and introduces new compounds may be effective in curing diseases and ailments^3^. Milk thistle, *Silybum marianum* (L.) Gaertn, a well-known medicinal plant belongs to Asteraceae family, is recently in center of attentions because of having a wide range therapeutic applications. Milk thistle is one of the most studied plant for treatment of liver disease due to having a group of flavonolignans called silymarin in its fruit integument. The hepatoprotective function of silymarin is mainly attributed to its anti-free radical and anticarcinogenic roles^4^ although but it includes other biological activities such as antioxidant, anti-lipid peroxidative, antifibrotic, anti-inflammatory, immunomodulatory and liver regenerating activity^5^. Silymarin has clinical applications in alcoholic liver diseases, liver cirrhosis, Amanita mushroom poisoning, viral hepatitis,toxic anddrugdiseases ofthe liver,psoriasis and in neuroprotective and neurotropic activity^5^. Silymarin acts as a toxin blockade agent by inhibiting binding of toxins to hepatocyte cell membrane receptors^6^.

Milk thistle contains 15–30% of triglycerides such as linoleic, oleic, and palmitic acids, and 30% of proteins, sugars (i.e., arabinose, galactose, glucose, fructose, rhamnose, sucrose, and xylose), sterols, tocopherol, and flavonoids (apigenin, chrysoeriol, dihydrokaempferol, eriodyctiol, kaempferol, naringin taxifolin, and quercetin)^7,8^. Lipid components, especially fatty acids, as high-value compounds used in food and pharmaceutical areas, play key roles in cell membranes structure and metabolic processes. Cell membranes maintenance, brain functions, transmission of nerve impulses, processes of transferring atmospheric oxygen to blood plasma, synthesis of hemoglobin, and cell division are the roles which are associated with Omega-series fatty acids in humans. Since human body is not able to synthesize these compounds, they have been known as curtail fatty acids^9^. Phytosterols, which mainly found in vegetables, fruits, nuts, and seeds, is another important dominant lipid component of this plant, which inhabits cholesterol absorption and is added to functional foods to increase their cholesterol-lowering ability^10^. Sitosterols, campesterols, and stigmasterols are the most extensive phytosterols in nature^11^. Beta-sitosterol (BS), one of phytosterols with a similar chemical structure to cholesterol, is synthesized in plants; whereas animals should acquire those through food diet^12^. BS possesses antimicrobial, antioxidant, immunomodulatory, angiogenic, anticancer, anti-inflammatory, antidiabetic, and antinociceptive activities without any major toxicity for human being^13^.

Global milk thistle products market has been growing at a CAGR of 7% from 2018 to 2022. Milk thistle one of top-selling herb supplements in the U.S. mass market, has introduced new opportunities such as food and feed consumption, bioenergy production, cosmetic and cosmeceutical applications, phytoremediation, industrial applications through the medium-large scale cultivation of this plant^14^.

Drought stress affects plant growth and development in many aspects, such as morphological, physiological, and phytochemical parameters, and reduces yield, dry biomass, and growth rate^15,16^.

Furthermore, composition of fatty acids and lipid content of organisms are affected by environmental conditions. In plants, biotic and abiotic factors affect primary and secondary metabolisms^17^. For example, drought conditions cause oxidative stress, which long chain fatty acids are transported from cytosol into mitochondrial membrane. Mitochondria break down sugar through citric acid cycle (CAC) and synthesize ATP to provide energy^18^. Oxygen is reduced to H_2_O at the end of CAC, leading to a reduction oxygen concentration and, subsequently, reducing reactive oxygen species (ROS) formation^19^. Therefore, plants ability to adapt to various environmental situations is affected by fatty acids and cell membrane lipids composition^20^.

Considering the importance of lipids, this research was aimed ^1^ to identify and measure bioactive compounds of milk thistle in field conditions under drought stress; (2) to identify genes involved in biosynthesis of these lipids and to determine interaction between genes and lipids; and finally, (3) to perform protein modeling and molecular docking of selected genes to confirm the role of these genes in stress tolerance.

## Material and methods

### Plant materials

Milk thistle seeds were obtained from the Pakanbazr, Isfahan, Iran. Plant study comply with relevant institutional, national, and international guidelines and legislation.

### Field experimental design and sampling

The experiment was established on Shahid Beheshti University’s experimental field in Tehran, Iran (51.23°N and 35.48°E) in a moderate and mountainous climate. The experiment was designed in a randomized complete block design (RCBD) with four replicates in three different drought stress levels [Field capacity (FC), 70% of FC and 40% of FC]. The soil texture consisted of 1/3 sand, 1/3 clay and 1/3 leaf composts. Average annual rainfall and temperature were 145.2 mm and of 22 °C, respectively. Average of air temperature, precipitation, relative humidity and wind speed were recorded monthly in meteorological site (Table 1). Field plot was 150.0 m^2^ (10 m wide and 15 m long). Seeds were sown every 0.5 m in the rows while 1 m distance was applied between the rows. Milk thistle seeds were purchased from the Pakanbazr Company, Isfahan, Iran. The seeds were sown on 16 March 2017, and irrigated every 2 days.

**Table 1:** Atmospheric information of experimental field (Iran, Tehran, Shemiranat) from meteorological site.

Atmospheric information	From 2017.03.21 to 2017.04.20	From 2017.04.21 to 2017.05.21	From 2017.05.22 to 2017.06.21	From 2017.06.22 to 2017.07.22
Average precipitation (mm)	60.5	76.7	0.0	26.3
Average temperature (°C)	12.7	19.6	25.8	28.1
Average moisture (%)	53	41	19	28
Average wind speed (mps)	10.0	17.0	12.0	10.0

Water deficit was applied to the plants at flowering stage on 24 June 2017. Weighing method was used to measure soil moisture. For this purpose, soil samples, three samples each day, were randomly taken from different areas of the field. Soil was irrigated just in first day and then did not irrigate until day six, when soil dried. Wet soils were weighted, and then oven-dried at 70 °C for 2 days. Dry weight of samples were measured. The difference between wet and dry weight showed the soil moisture. The soil moisture content was considered as 100% for field capacity, the moisture content for treatments with 70% and 40% FC were calculated accordingly. Therefore, irrigation was done every two days for FC, every 4 days for 70% FC, and every 6 days for 40% FC irrigation was performed^15^. Four plants were randomly selected in each group, and their seeds were collected and dried under shade condition in the laboratory for a month. Finally, dried seed were used for extraction and further analysis.

### Isolation of oil and methanolic extract and GC–MS analysis

Dried seed were completely milled into powders. To isolate oil extract, 10 g of seed powder was used in a Soxhlet extractor in presence of n-hexane solvent. The extraction was done at 70 °C for 6 h and the final extract was stored in a dark glass. Oil-free powder was incubated at 37 °C for a week to dry, and then used for methanolic extraction^21^.

To isolate methanol extract, 2 g of oil-free powder was mixed with 200 ml of 80% methanol on a shaker for two days. The mixture was then passed through a filter paper and then stored at 4 °C. The same procedure was repeated for remaining powder on the filter paper and resulted extract was added into the first collected portion. Finally, the extracts were exposed to room temperature to be concentrated for 2 weeks^22^.

GC–MS analysis were performed using a Trace Gas Chromatograph 2000 Series supplied with a Finnigan Trace mass spectrometer, using helium as carrier gas (36.445 cm/s), supplied with a DB-1 J&W capillary column (30 m × 0.25 mm i.d.,0.25 mm film thickness). The chromatographic conditions were followed by an initial temperature at 70 °C for 5 min, a temperature rate: 5 °C/min, and a final temperature at 290 °C for 10 min. Injector temperature was 300 °C, transfer-line temperature was 300 °C with a split ratio of 20:1. The extracts were dissolved in n-hexane solvent separately and 1–2 µl of each extracts was injected into apparatus. GC–MS data and peaks were analyzed, components of each extract were identified and their amounts were measured.

### Bioinformatics analysis

Kyoto Encyclopedia of Genes and Genomes (KEGG) (https://www.genome.jp/kegg/)^23^ and Metabolic Pathways From all Domains of Life (MetaCyc) (https://metacyc.org/)^24^ databases were used to identify biosynthesis pathways as well as enzymes and genes involved in synthesis of extracts components. For this purpose, the desired lipids name was searched through KEGG and MetaCyc databases and in the "Compounds" section, the desired lipid was selected and the required information was extracted. The genes involved in the synthesis of these lipids were selected from the Arabidopsis model plant because most of its genes are known. GENEVESTIGATOR-Visualizing the world's expression data software (https://genevestigator.com/)^25^ was applied to investigate expression of genes under drought stress. Then Biclustering tool was used to compare the expression level of selected genes in different Arabidopsis datasets. Functional protein association networks (STRING) (https://string-db.org/)^26^ and ShinyGO v0.61 programs (http://bioinformatics.sdstate.edu/go/)^27^ were used to determine proteins interaction and biology processes interactions, respectively. ANOVA analysis for all measured lipids were performed using R version 3.5.3 (https://www.r-project.org/)^28^ and RStudio version 1.1.463 (https://www.rstudio.com/)^29^. Data were analyzed in a completely randomized blocks design (CRBD) with treatments as fixed effects and replications as random effect. Mean comparison was calculated by Duncan test presented in the agricolae package at 5% significance level of probability^30^. Box plot to represent relative gene expression was also drawn by R software.

### Molecular analysis

#### RNA extraction and cDNA synthesis

Total RNA was extracted from 0.2 g mature seeds of milk thistle in the flowering stage using a total RNA kit (RB1001, RNA, Iran) and subsequently treated with DNase I (RB125A, RNA, Iran) to eliminate probable genomic DNA contamination. The quality and quantity of the extracted RNA were assessed with 1% agarose gel and NanoDrop 1000 spectrophotometer (Thermo Scientific, USA), respectively. Purified RNA (Concentration 5 µg) was used to synthesize first-strand cDNA through cDNA Synthesis kit (RB125A, RNA, Iran) following the manufacturer’s protocola, and stored at − 20 °C until use.

#### Primer design, sequence submit and qRT-PCR analysis

To design primers of the genes of interest, nucleotide sequences of same family plants (*Helianthus annuus* and *Carthamus tinctorius*) were obtained from the NCBI database. These sequences were then blasted on the milk thistle assembly data (from our previous experiment) using the BlastStation tool and the most identity sequences were selected. To primer design for qPCR, areas near to end of poly adenine, with a length of 150–250 bp were chosen. Homodimer, heterodimer, stem-loop, GC percent, and TM temperature were evaluated using Oligo 7 software (https://www.oligo.net/)^31^ and Vector NTI^®^ Express Designer Software (https://vector-nti.software.informer.com/11.0/)^16^. The 18SrRNA gene was used as the reference gene. Finally, primers were synthesized by Bioneer Company (South Korea). The sequence and other information of primers are listed in Table 2.

**Table 2:** RT-qPCR primer sequence of selected candidate and reference genes and their amplification characteristics.

Primer name	Primer sequence	PCR product length (bp)	TM	PCR amplification efficiency
*PXG3*	F: CCAGCAAACCTTGAGAAC	207	54	1.95
	R: GCAACGCCTTACTGATTC		54	
*PAL*	F: AGGGTAATCTAATAGGCC	155	52	1.88
	R: ACTGAACTCTCCATCTGG		54	
*LOX2*	F: CCACAGTGGAAACATGTC	255	54	1.94
	R: ATCTTCAACCGCCATACC		54	
*CYP710A1*	F: TTCTACCTACACTGAGC	198	50	1.90
	R: AGGAAGTCAAACAGGTGG		54	
*PLA2-ALPHA*	F: ATGGGAAGTACTGTGGG	194	52	1.99
	R: GTGTTGCCTTTGAATGTC		52	
*LACS3*	F: GAGATGAATTATGACGCC	256	52	1.91
	R: GCCACATATTCTCCTTG		50	
*CYP86A1*	F: ACGTGACACCTCCTCCG	242	57	1.97
	R: CATGTTCGTGGTCCAGCG		58	
*FATA2*	F: GTACTAGACGTGATTGG	177	50	2.00
	R: CTTCTGGAAATGCTAATC		49	
*18SrRNA*	F:ATGATAACTCGACGGATCGC	200	56	1.97
	R:CTTGGATGTGGTAGCCGTTT		57	

For sequence submit of selected genes on NCBI site, the CDS format of selected sequences in milk thistle was obtained using Vector NTI software. Finally, to confirm the results, these sequences were blasted at the NCBI site, resulting in sequences of these genes in other plants.

RT-qPCR amplification was carried out by Rotor-Gene 2000 (Corbett Life Science, Sydney, Australia) using SYBR^®^ Green Real-Time PCR Master Mix (RB120, RNA, Iran). 20 µl reactions included 10 μL 2 × SYBR, 1 μL of synthesized cDNA, 1 μL of each primer (20 nmol), and 7 μL RNase-free water. The thermal conditions consisted of an initial step for 5 min at 95 °C, followed by 35 cycles amplification (1 min at 95 °C, 1 min at 50–60 °C depending on primers tm, and 15 s at 72 °C). To investigate the specificity of each amplicon, post-amplification melting-curve ranging from 60 to 95 °C were assessed in every reaction. Cycle threshold values and PCR efficiency were computed by LinRegPCR program (https://www.gear-genomics.com/rdml-tools/)^32^, and relative expression levels were calculated by Relative Expression Software Tool (REST) (https://www.gene-quantification.de/rest.html)^33^ with the following formula. The E in the equation refers to the primer efficiency. All the qRT-PCR analysis were done in three biological and three technical replicates.

$$Gene\, expression\, ratio={({E}_{Gene})}^{\Delta\, ct\, Gene}/{({E}_{Ref})}^{\Delta\, ct\, Ref}.$$

### Protein analysis

Vector NTI software was used to identify ORFs of selected gene sequences, which were later translated into protein sequences using the SIB Swiss Institute of Bioinformatics (Expasy) (https://www.expasy.org/)^34^. Homology modeling was implemented to obtain the 3D (three-dimensional) structures of these proteins using trRosetta (https://yanglab.nankai.edu.cn/trRosetta/)^35^. HHsearch against the PDB70 database was used to detect template in trRosetta server. TM-score of the predicted models was estimated based on the probability of top predicted distance and convergence of top models. Next, 3D structures of proteins were used to create Ramachandran plot by ProFunc server (http://www.ebi.ac.uk/thornton-srv/databases/ProFunc/)^36^. Proteins functionality were predicted by Protein Structure Classification Database at UCL (CATH) (https://www.cathdb.info/)^37^. Molecular docking was performed by protein–ligand binding site prediction (COACH-D) (https://yanglab.nankai.edu.cn/COACH-D/)^38^ using 3D models as inputs. Atomistic interactions between proteins and predicted ligands were studied within 5A of ligand residues using VMD-Visual Molecular Dynamics software (https://www.ks.uiuc.edu/Research/vmd/)^39^. Hydrogen bonds between proteins and ligands were identified using PyMOL software (https://pymol.org/2/)^40^. All modeled structures also were visualized though PyMOL.

## Results

### Identification of oil and methanolic extracts compounds

**Figure 1:**
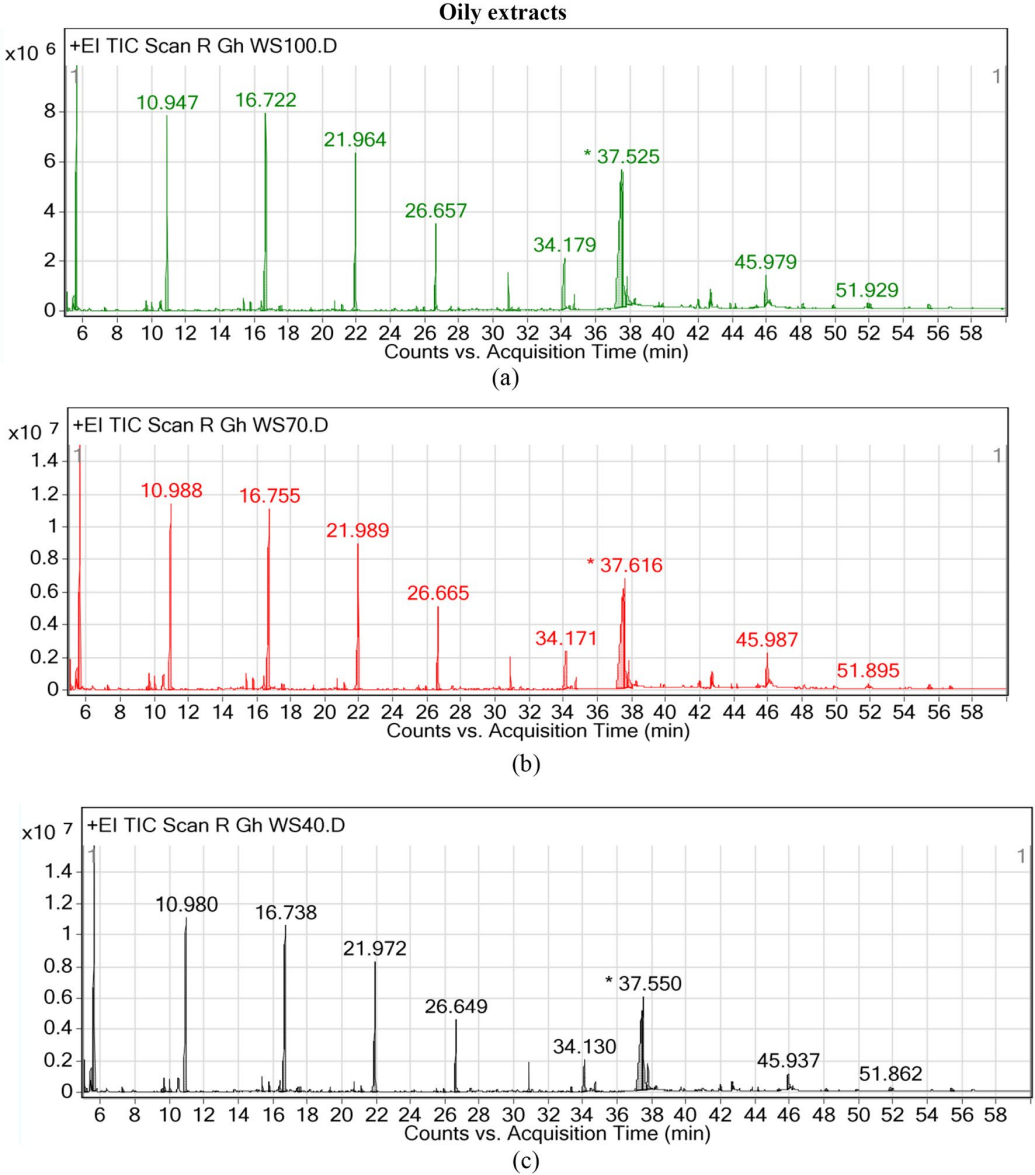
GC–MS peaks of oily extracts in milk thistle seeds under three different drought stress levels. (**a**) Field capacity: 100% FC, (**b**) 70% FC, (**c**) 40% FC.

**Figure 2:**
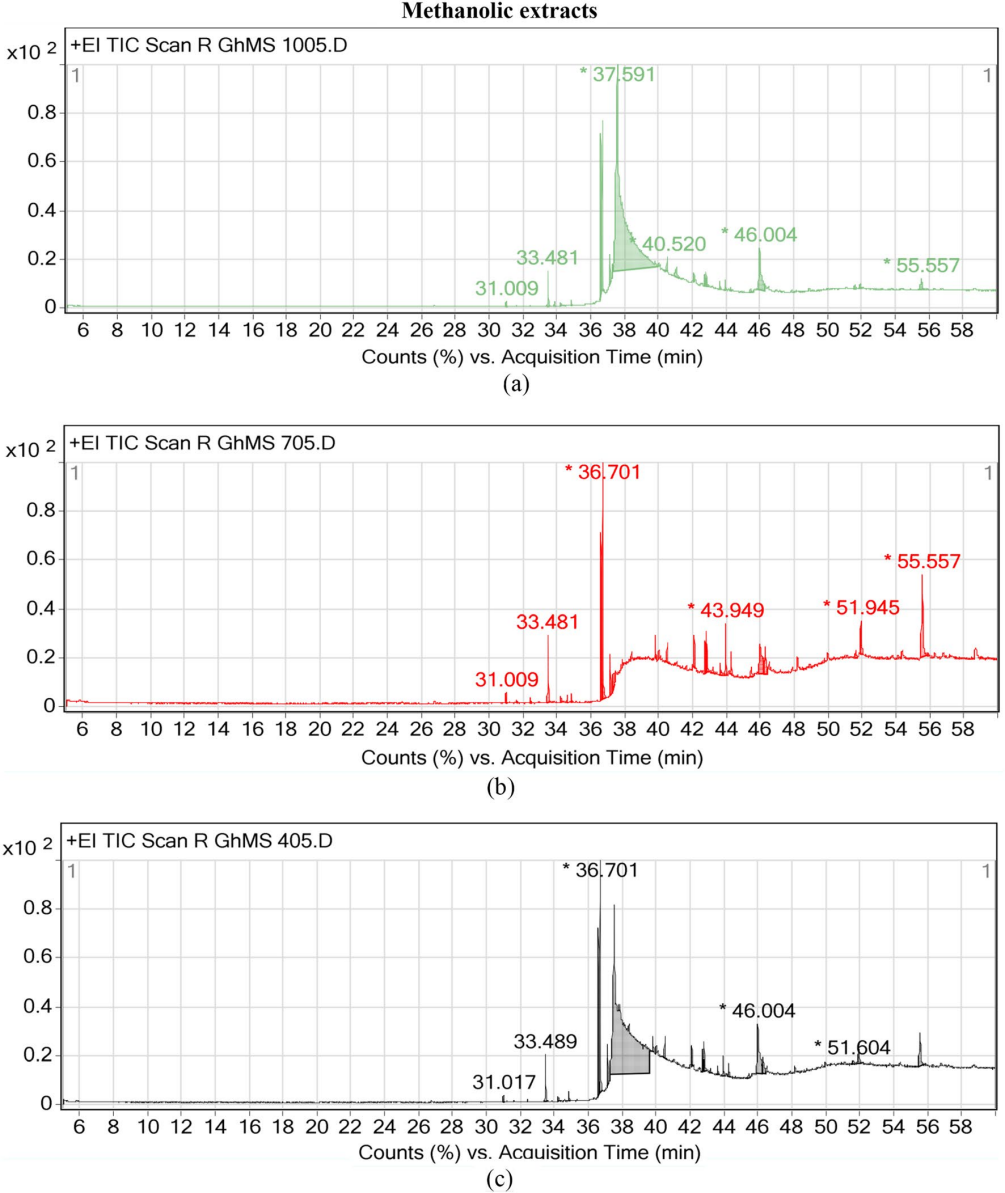
GC–MS peaks of methanolic extracts in milk thistle seeds under three different drought stress levels. (**a**) Field capacity: 100% FC, (**b**) 70% FC, (**c**) 40% FC.

12 compounds in oil extract and 13 compounds in methanolic extract of milk thistle seeds were identified by analysis of GC–MS peaks (Figs. 1 and 2). Name, chemical formula, category, biosynthetic pathways and reactions, enzymes and genes involved in the synthesis of this components were listed in Table 3. In spite of the fact that many genes in milk thistle have not yet been known, *Arabidopsis thaliana* has been used as a model plant to determine genes, pathways and reactions.

**Table 3:** Milk thistle lipids and their category, pathways, reactions, enzymes, genes and definitions.

Components	Category	Pathways	Reactions	Enzymes	Genes	Definition
**Oil extract**						
Tetradecane (C14H30)	Hydrocarbon	Alkane biosynthesis	A long-chain fatty acid + hν + H^+^ → a long-chain alkane + CO2	Fatty acid photodecarboxylase	AT3G50700	
Hexadecane (C16H34)		Alkane biosynthesis	A long-chain fatty acid + hν + H^+^ → a long-chain alkane + CO2	Fatty acid photodecarboxylase	AT3G50700	
Octadecane (C18H38)		Alkane biosynthesis	A long-chain fatty acid + hν + H^+^ → a long-chain alkane + CO2	Fatty acid photodecarboxylase	AT3G50700	
3-Methyl-heptane (C8H18)		Alkane biosynthesis	A long-chain fatty aldehyde + 2 NADPH+ oxygen + H^+^ → an alkane + formate + 2 NADP + + H2O	Aldehyde decarbonylase	D64155.1	
Octane (C8H18)		Alkane biosynthesis	Octane + 2 reduced rubredoxin + oxygen + 2 H^+^ <=> 1-octanol + 2 oxidized rubredoxin + H2O	Alkane 1-monooxygenase	D64155.1	
Decane (C10H22)		Alkane biosynthesis	A long-chain fatty aldehyde + 2 NADPH+ oxygen + H^+^ → an alkane + formate + 2 NADP + + H2O	Aldehyde decarbonylase	D64155.1	
Dodecane (C12H26)		Alkane biosynthesis	A long-chain fatty aldehyde + 2 NADPH+ oxygen + H^+^ → an alkane + formate + 2 NADP + + H2O	Aldehyde decarbonylase	D64155.1	
Stearic acid (C18H35O2)	Fatty acid	Fatty acid biosynthesis Biosynthesis of unsaturated fatty acids Biosynthesis of plant secondary metabolites Phototransduction—fly	Octadecanoyl-[acyl-carrier protein] + H2O <=> Acyl-carrier protein + Octadecanoic acid Stearoyl-CoA + H2O <=> CoA + octadecanoic acid ATP + octadecanoic acid <=> diphosphate + (stearoyl)adenylate ATP + octadecanoic acid + holo-[(hydroxy)phthioceranic acid synthase] <=> AMP + diphosphate + stearoyl-[(hydroxy)phthioceranic acid synthase]	Palmitoyl-CoA hydrolase Oleoyl-[acyl-carrier-protein] hydrolase Long-chain fatty acid adenylase/transferase FadD23	AT4G00520	Acyl-CoA thioesterase family protein
					AT1G01710	Acyl-CoA thioesterase II
					AT1G08510	Fatty acyl-ACP thioesterases B
					AT3G25110	FatA acyl-ACP thioesterase
					AT4G13050	Acyl-ACP thioesterase
Palmitic acid (C16H31O2)		Fatty acid biosynthesis Fatty acid elongation Fatty acid degradation Cutin, suberine and wax biosynthesis Biosynthesis of unsaturated fatty acids Biosynthesis of plant secondary metabolites Metabolic pathways Fatty acid metabolism	Palmitoyl-CoA + H2O <=> CoA + hexadecanoic acid ATP + hexadecanoic acid + CoA <=> AMP + palmitoyl-CoA + diphosphate Hexadecanoic acid + 2 hydrogen peroxide <=> pentadecanal + CO2 + 3 H2O Hexadecanal + NAD+ + H2O <=> hexadecanoic acid + NADH+ H+ Hexadecanoic acid + protein <=> palmitoyl-protein + H2O Hexadecanoyl-[acp] + H2O <=> acyl-carrier protein + hexadecanoic acid Retinyl palmitate + H2O <=> retinol + hexadecanoic acid 11-cis-Retinyl palmitate + H2O <=> 11-cis-retinol + hexadecanoic acid *S*-Palmitoylprotein + H2O <=> hexadecanoic acid + [protein]-l-cysteine Hexadecanoic acid + [reduced NADPH---hemoprotein reductase] + oxygen <=> 16-Hydroxypalmitate + [oxidized NADPH---hemoprotein reductase] + H2O ATP + hexadecanoic acid <=> diphosphate + (palmitoyl)adenylate ATP + hexadecanoic acid + holo-[(hydroxy)phthioceranic acid synthase] <=> AMP + diphosphate + palmitoyl-[(hydroxy)phthioceranic acid synthase]	Long-chain-aldehyde dehydrogenase Fatty-acid peroxidase Long-chain fatty acid omega-monooxygenase Fatty-acid synthase system 11-cis-retinyl-palmitate hydrolase Palmitoyl-CoA hydrolase Oleoyl-[acyl-carrier-protein] hydrolase Palmitoyl[protein] hydrolase Long-chain-fatty-acid---CoA ligase Long-chain fatty acid adenylase/transferase FadD23	AT1G69500	Cytochrome P450, family 704, subfamily B, polypeptide 1
					AT5G58860	Cytochrome P450, family 86, subfamily A, polypeptide 1
					AT1G01710	Acyl-CoA thioesterase II
					AT4G00520	Acyl-CoA thioesterase family protein
					AT1G08510	Fatty acyl-ACP thioesterases B
					AT3G25110	FatA acyl-ACP thioesterase
					AT4G13050	Acyl-ACP thioesterase
					AT1G13610	alpha/beta-Hydrolases superfamily protein
					AT1G32190	Alpha/beta-hydrolases superfamily protein
					AT1G66900	Alpha/beta-hydrolases superfamily protein
					AT2G24320	Alpha/beta-hydrolases superfamily protein
					AT3G01690	Alpha/beta-hydrolases superfamily protein
					AT3G30380	Alpha/beta-hydrolases superfamily protein
					AT3G60340	Alpha/beta-hydrolases superfamily protein
					AT4G17470	Alpha/beta-hydrolases superfamily protein
					AT4G17480	Alpha/beta-hydrolases superfamily protein
					AT4G17483	Alpha/beta-hydrolases superfamily protein
					AT4G24760	Alpha/beta-hydrolases superfamily protein
					AT4G31020	Alpha/beta-hydrolases superfamily protein
					AT5G14390	Alpha/beta-hydrolases superfamily protein
					AT5G38220	Alpha/beta-hydrolases superfamily protein
					AT5G47330	Alpha/beta-hydrolases superfamily protein
					AT5G47340	Alpha/beta-hydrolases superfamily protein
					AT5G47350	Alpha/beta-hydrolases superfamily protein
					AT1G49430	Long-chain acyl-CoA synthetase 2(LACS2)
					AT1G64400	AMP-dependent synthetase and ligase family protein(LACS3)
					AT1G77590	Long chain acyl-CoA synthetase 9(LACS9)
					AT2G04350	AMP-dependent synthetase and ligase family protein(LACS8)
					AT2G47240	AMP-dependent synthetase and ligase family protein(LACS1)
					AT3G05970	Long-chain acyl-CoA synthetase 6(LACS6)
					AT3G23790	AMP-dependent synthetase and ligase family protein(AAE16)
					AT4G11030	AMP-dependent synthetase and ligase family protein
					AT4G14070	Acyl-activating enzyme 15(AAE15)
					AT4G23850	AMP-dependent synthetase and ligase family protein(LACS4)
					AT5G27600	Long-chain acyl-CoA synthetase 7(LACS7)
Linoleic acid (C18H31O2)		Linoleic acid metabolism Biosynthesis of unsaturated fatty acids Biosynthesis of plant secondary metabolites Metabolic pathways	Linoleate + oxygen <=> (9Z,11E)-(13S)-13-hydroperoxyoctadeca-9,11-dienoic acid Linoleate <=> rumenic acid Linoleate + oxygen <=> (9Z,12Z)-(11S)-11-hydroperoxyoctadeca-9,12-dienoic acid Linoleate + 2 ferrocytochrome b5 + oxygen + 2 H+ <=> crepenynate + 2 ferricytochrome b5 + 2 H2O Linoleate + oxygen + NADPH+ H+ <=> 9(10)-EpOME + NADP + + H2O Linoleate + oxygen + NADPH+ H+ <=> 12(13)-EpOME + NADP + + H2O Linoleate + oxygen <=> 9(S)-HPODE Linoleate + oxygen <=> 8(R)-HPODE Linoleate + reduced acceptor + oxygen <=> (6Z,9Z,12Z)-octadecatrienoic acid + acceptor + 2 H2O Phosphatidylcholine + H2O <=> 1-acyl-sn-glycero-3-phosphocholine + linoleate Linoleoyl-CoA + H2O <=> CoA + linoleate Linoleate + oxygen <=> (8E,10R,12Z)-10-hydroperoxy-8,12-octadecadienoate Linoleate + oxygen <=> (8E,10S,12Z)-10-hydroperoxyoctadeca-8,12-dienoate	Linoleate 13S-lipoxygenase Arachidonate 15-lipoxygenase Linoleate 11-lipoxygenase Linoleate 9S-lipoxygenase Linoleate 8R-lipoxygenase Linoleate 10R-lipoxygenase Oleate 10S-lipoxygenase Unspecific monooxygenase Acyl-CoA 6-desaturase Acyl-lipid Delta12-acetylenase Phospholipase A2 Palmitoyl-CoA hydrolase Linoleate isomerase	AT1G17420	Lipoxygenase 3(LOX3)
					AT1G67560	PLAT/LH2 domain-containing lipoxygenase family protein(LOX6)
					AT1G72520	PLAT/LH2 domain-containing lipoxygenase family protein(LOX4)
					AT3G45140	lipoxygenase 2(LOX2)
					AT1G55020	lipoxygenase 1(LOX1)
					AT3G22400	PLAT/LH2 domain-containing lipoxygenase family protein(LOX5)
					AT2G06925	Phospholipase A2 family protein(PLA2-ALPHA)
					AT3G57140	sugar-dependent 1-like protein(SDP1-LIKE)
					AT5G04040	Patatin-like phospholipase family protein(SDP1)
					AT1G01710	acyl-CoA thioesterase II
					AT4G00520	Acyl-CoA thioesterase family protein
cis-13-Octadecenoic acid (C18H34O2)		Cutin biosynthesis, oleate biosynthesis II (animals and fungi), sporopollenin precursors biosynthesis, suberin monomers biosynthesis	Oleoyl-CoA + H2O → oleate + coenzyme A + H+	Oleoyl-CoA thioesterase	AT2G23390	
1-Oleoyl-glycerol (C21H40O4)	Ester	triacylglycerol degradation	A 1,2-diacyl-sn-glycerol + H2O → a 2-acylglycerol + a fatty acid + H+	Sn1-specific diacylglycerol lipase	AT1G05790	
**Methanolic extract**						
Methyl linoleate (C19H34O2)	Fatty acid	Acyl-CoA hydrolysis	A 2,3,4-saturated fatty acyl CoA + H2O → a 2,3,4-saturated fatty acid + coenzyme A + H+	Acyl-CoA thioesterase	AT2G23390	
Methyl stearate (C19H38O2)		Acyl-CoA hydrolysis	A 2,3,4-saturated fatty acyl CoA + H2O → a 2,3,4-saturated fatty acid + coenzyme A + H+	Acyl-CoA thioesterase	AT2G23390	
Methyl 9-octadecenoate (C19H36O2)		Acyl-CoA hydrolysis	A 2,3,4-saturated fatty acyl CoA + H2O → a 2,3,4-saturated fatty acid + coenzyme A + H+	Acyl-CoA thioesterase	AT2G23390	
Methyl palmitate (C17H34O2)		Acyl-CoA hydrolysis	A 2,3,4-saturated fatty acyl CoA + H2O → a 2,3,4-saturated fatty acid + coenzyme A + H+	Acyl-CoA thioesterase	AT2G23390	
Oleic acid (C18H33O2)		Fatty acid biosynthesis; cutin, suberine and wax biosynthesis; biosynthesis of unsaturated fatty acids; biosynthesis of plant secondary metabolites; longevity regulating pathway—worm	(9Z)-octadecenoic acid + oxygen <=> (8E,10S)-10-hydroperoxyoctadeca-8-enoate Oleamide + H2O <=> (9Z)-octadecenoic acid + ammonia (9Z)-octadecenoic acid + lipid hydroperoxide <=> cis-9,10-epoxystearic acid + alcohol (9Z)-octadecenoic acid + [reduced NADPH---hemoprotein reductase] + oxygen <=> 18-hydroxyoleate + [oxidized NADPH---hemoprotein reductase] + H2O Oleoyl-CoA + H2O <=> CoA + (9Z)-octadecenoic acid Oleoyl-[acyl-carrier protein] + H2O <=> acyl-carrier protein + (9Z)-octadecenoic acid (R)-10-hydroxystearate <=> (9Z)-octadecenoic acid + H2O	Plant seed peroxygenase; plant peroxygenase, soybean peroxygenase Oleate 10S-lipoxygenase Long-chain fatty acid omega-monooxygenase Palmitoyl-CoA hydrolase Oleoyl-[acyl-carrier-protein] hydrolase Fatty acid amide hydrolase Oleate hydratase	AT1G23240	Caleosin-related family protein
					AT1G70670	Caleosin-related family protein
					AT1G70680	Caleosin-related family protein
					AT2G33380	Caleosin-related family protein
					AT4G26740	peroxygenase 1
					AT5G29560	caleosin-related family protein
					AT5G55240	PEROXYGENASE 2 (ATPXG2)
					AT1G69500	cytochrome P450, family 704, subfamily B, polypeptide 1(CYP704B1)
					AT5G58860	cytochrome P450, family 86, subfamily A, polypeptide 1(CYP86A1)
					AT1G01710	acyl-CoA thioesterase II
					AT4G00520	Acyl-CoA thioesterase family protein
					AT1G08510	fatty acyl-ACP thioesterases B(FATB)
					AT3G25110	fatA acyl-ACP thioesterase(FaTA)
					AT4G13050	Acyl-ACP thioesterase
β-Monolinolein (C21H38O4)		Linoleate biosynthesis	A [glycerolipid]-oleate + 2 a reduced ferredoxin [iron-sulfur] cluster + oxygen + 2 H+ → a [glycerolipid]-linoleate + 2 an oxidized ferredoxin [iron-sulfur] cluster + 2 H2O	Acyl-lipid ω-6 desaturase (ferredoxin)	AT4G30950	
Methyl behenate (C23H46O2)	Ester	–	S-adenosyl-l-methionine + a fatty acid → S-adenosyl-l-homocysteine + a fatty acid-methyl ester	–	–	–
Linoleic acid ethyl ester (C20H34O2)		–	A carboxylic ester + H2O → an alcohol + a carboxylate + H+	Carboxylesterase 3	AT4G22300	
Glycerol β-stearate (C21H42O4)		Triacylglycerol degradation	A 1,2-diacyl-sn-glycerol + H2O → a 2-acylglycerol + a fatty acid + H+	Triacylglycerol lipase	AT1G45201	
Phthalic acid, dioctyl ester (C24H38O4)		–	Bis(2-ethylhexyl)phthalate + H2O → 2-ethylhexan-1-ol + 2-ethylhexyl phthalate + H+	–	–	
Hexadecanoic acid, 2-(octadecyloxy) ethyl ester (C36H72O3)		–	A carboxylic ester + H2O → an alcohol + a carboxylate + H+	Carboxylesterase 3	AT4G22300	
Cholesterol (C27H46O)	Steroid	Steroid biosynthesis Steroid hormone biosynthesis Steroid degradation Biosynthesis of alkaloids derived from terpenoid and polyketide Metabolic pathways Fat digestion and absorption Vitamin digestion and absorption Cholesterol metabolism	Cholesterol + NAD+ <=> 7-dehydrocholesterol + NADH+ H+ Cholesterol + oxygen + NADPH+ H+ <=> cholesterol-5alpha,6alpha-epoxide + NADP + + H2O Cholesterol + oxygen + NADPH+ H+ <=> cholesterol-5beta,6beta-epoxide + NADP + + H2O Cholesterol + Oxygen + 2 H+ + 2 Reduced adrenal ferredoxin <=> 20alpha-Hydroxycholesterol + H2O + 2 Oxidized adrenal ferredoxin Cholesterol + NADP + <=> 7-Dehydrocholesterol + NADPH+ H+ Cholesterol + NADP + <=> desmosterol + H+ + NADPH Cholesterol + oxygen <=> cholest-4-en-3-one + hydrogen peroxide Cholesteryl-beta-D-glucoside + H2O <=> cholesterol + D-glucose Acyl-CoA + cholesterol <=> CoA + cholesterol ester Cholesterol ester + H2O <=> cholesterol + fatty acid Cholesterol + oxygen + [reduced NADPH---hemoprotein reductase] <=> 7alpha-hydroxycholesterol + [oxidized NADPH---hemoprotein reductase] + H2O 1,2-Diacyl-sn-glycerol + cholesterol <=> 1-acylglycerol + cholesterol ester Cholesterol + oxygen + 2 reduced adrenal ferredoxin + 2 H+ <=> 22(R)-hydroxycholesterol + H2O + 2 oxidized adrenal ferredoxin Cholesterol + 3 oxygen + 6 H+ + 6 reduced adrenal ferredoxin <=> 4-methylpentanal + pregnenolone + 4 H2O + 6 oxidized adrenal ferredoxin Cholesterol + [reduced NADPH---hemoprotein reductase] + oxygen <=> cerebrosterol + [oxidized NADPH---hemoprotein reductase] + H2O Cholesterol + reduced acceptor + oxygen <=> 25-hydroxycholesterol + acceptor + H2O Cholesterol + oxygen + 2 H+ + 2 reduced adrenal ferredoxin <=> cholest-5-ene-3beta,26-diol + H2O + 2 oxidized adrenal ferredoxin Cholesterol + sulfate <=> cholesterol sulfate + H2O Cholesterol + 3'-phosphoadenylyl sulfate <=> cholesterol sulfate + adenosine 3',5'-bisphosphate Cholesterol + NAD+ <=> cholest-4-en-3-one + NADH+ H+ Cholesterol + oxygen + NADH+ H+ <=> 7-dehydrocholesterol + NAD+ + 2 H2O Cholesterol + oxygen + NADPH+ H+ <=> 7-Dehydrocholesterol + NADP + + 2 H2O	3Beta-hydroxy-Delta5-steroid dehydrogenase Cholesterol oxidase 7-dehydrocholesterol reductase Delta24-sterol reductase cholesterol 7alpha-monooxygenase Cholesterol 24-hydroxylase Cholesterol monooxygenase (side-chain-cleaving) Cholestanetriol 26-monooxygenase Cholesterol 7-desaturase Cholesterol 25-hydroxylase Sterol O-acyltransferase Diacylglycerol---sterol O-acyltransferase Alcohol sulfotransferase Bile-salt sulfotransferase Sterol esterase Steryl-sulfatase Steryl-beta-glucosidase Steroid Delta-isomerase	AT1G50430	Ergosterol biosynthesis ERG4/ERG24 family(DWF5)
					AT3G19820	cell elongation protein / DWARF1 / DIMINUTO (DIM) (DWF1)
					AT3G57140	sugar-dependent 1-like protein(SDP1-LIKE)
					AT5G04040	Patatin-like phospholipase family protein(SDP1)
β-Sitosterol (C29H50O)	Lipid	Steroid biosynthesis Biosynthesis of plant secondary metabolites Biosynthesis of terpenoids and steroids Biosynthesis of secondary metabolites	Isofucosterol + NADPH+ H+ <=> beta-sitosterol + NADP + beta-Sitosterol + NADPH+ H+ + oxygen <=> stigmasterol + NADP + + 2 H2O	Sterol 22-desaturase	AT2G28850	cytochrome P450, family 710, subfamily A, polypeptide 3(CYP710A3)
					AT2G28860	cytochrome P450, family 710, subfamily A, polypeptide 4(CYP710A4)
					AT2G34490	cytochrome P450, family 710, subfamily A, polypeptide 2(CYP710A2)
					AT2G34500	cytochrome P450, family 710, subfamily A, polypeptide 1(CYP710A1)

Oil extract contained hydrocarbons, fatty acids and esters. Hydrocarbons included Tetradecane, Hexadecane, Octadecane, 3-methyl-Heptane, Octane, Decane and Dodecane, which were active in alkane biosynthesis pathway. The enzymes involved in these pathways were fatty acid photodecarboxylase, aldehyde decarbonylase and alkane 1-monooxygenase.

The fatty acids included Stearic acid, Palmitic acid, Linoleic acid and cis-13-Octadecenoic acid. Stearic acid was produced in biosynthesis pathways of fatty acid and secondary metabolites. Enzymes involved in mentioned biosynthesis pathways were palmitoyl-CoA hydrolase, oleoyl hydrolase and long-chain fatty acid adenylase/transferase. Palmitic acid was involved in fatty acids and secondary metabolites biosynthesis pathways, elongation, degradation and fatty acids metabolism as well as cutin, suberine and wax biosynthesis pathways. Fatty acid aldehyde dehydrogenase, peroxidase, omega-monooxygenase, fatty acid synthase, oleoyl hydrolase, CoA ligase, adenylase/transferase FadD23, 11-cis-retinyl-palmitate hydrolase and palmitoyl-CoA hydrolase were engaged in biosynthesis pathways. Linoleic acid was also engaged in fatty acids and secondary metabolites biosynthesis as well as linoleic acid metabolism pathways. Linoleate lipoxygenase family enzymes were predominant, which catalyzes reaction between linoleate and oxygen to produce hydroperoxyoctadeca dienoic acid (HPODE). Cis-13-octadecenoic acid fatty acid was involved in cutin, oleate, sporopollenin precursors, suberin monomers biosynthesis and catalyzed by oleoyl-CoA thioesterase. The only identified ester was 1-oleoyl-glycerol, which was involved in triacylglycerol degradation by sn1-specific diacylglycerol lipase activity.

Methanolic extract contained other fatty acids, esters, steroids and lipids. Fatty acids included Methyl linoleate, Methyl stearate, Methyl 9-octadecenoate, Methyl palmitate, Oleic acid and β-Monolinolein. The enzymes involved in these pathways were peroxygenase, lipoxygenase, monooxygenase, hydrolase and hydratase. Methylated fatty acids were synthesized by thioesterase acyl-CoA enzyme in acyl-CoA hydrolysis pathway. Oleic acid, the major fatty acid in methanolic extract, was active in biosynthesis pathways of fatty acids, cutin, suberine, wax, and plant secondary metabolites. β-Monolinolein fatty acid was responsible in linoleate biosynthesis pathway through acyl-lipid ω-6 desaturase (ferredoxin) activity.

Esters comprised Methyl behenate, Linoleic acid ethyl ester, Glycerol β-stearate, Phthalic acid dioctyl ester and Hexadecanoic acid 2-(octadecyloxy) ethyl ester, which the engaged enzymes were carboxylesterase 3 and triacylglycerol lipase, which were active in triacylglycerol degradation pathway. Cholesterol belonged to steroids, which were active in steroid hormone biosynthesis, steroid biosynthesis and degradation, biosynthesis of alkaloids derived from terpenoid and polyketide, fat and vitamin digestion and absorption, cholesterol metabolism and metabolic pathways by involving 3beta-hydroxy-Delta5-steroid dehydrogenase, cholesterol oxidase, monooxygenase, hydroxylase and desaturase, 7-dehydrocholesterol and Delta24-sterol reductase, sterol acyltransferase and esterase, alcohol and bile-salt sulfotransferase, steryl sulfatase and glucosidase, steroid Delta-isomerase. The identified lipid was β-Sitosterol, which was synthesized by engaging sterol 22-desaturase enzymes in steroid, plant secondary metabolites and terpenoids biosynthesis pathways.

### Gene expression analysis using Genevestigator

**Figure 3:**
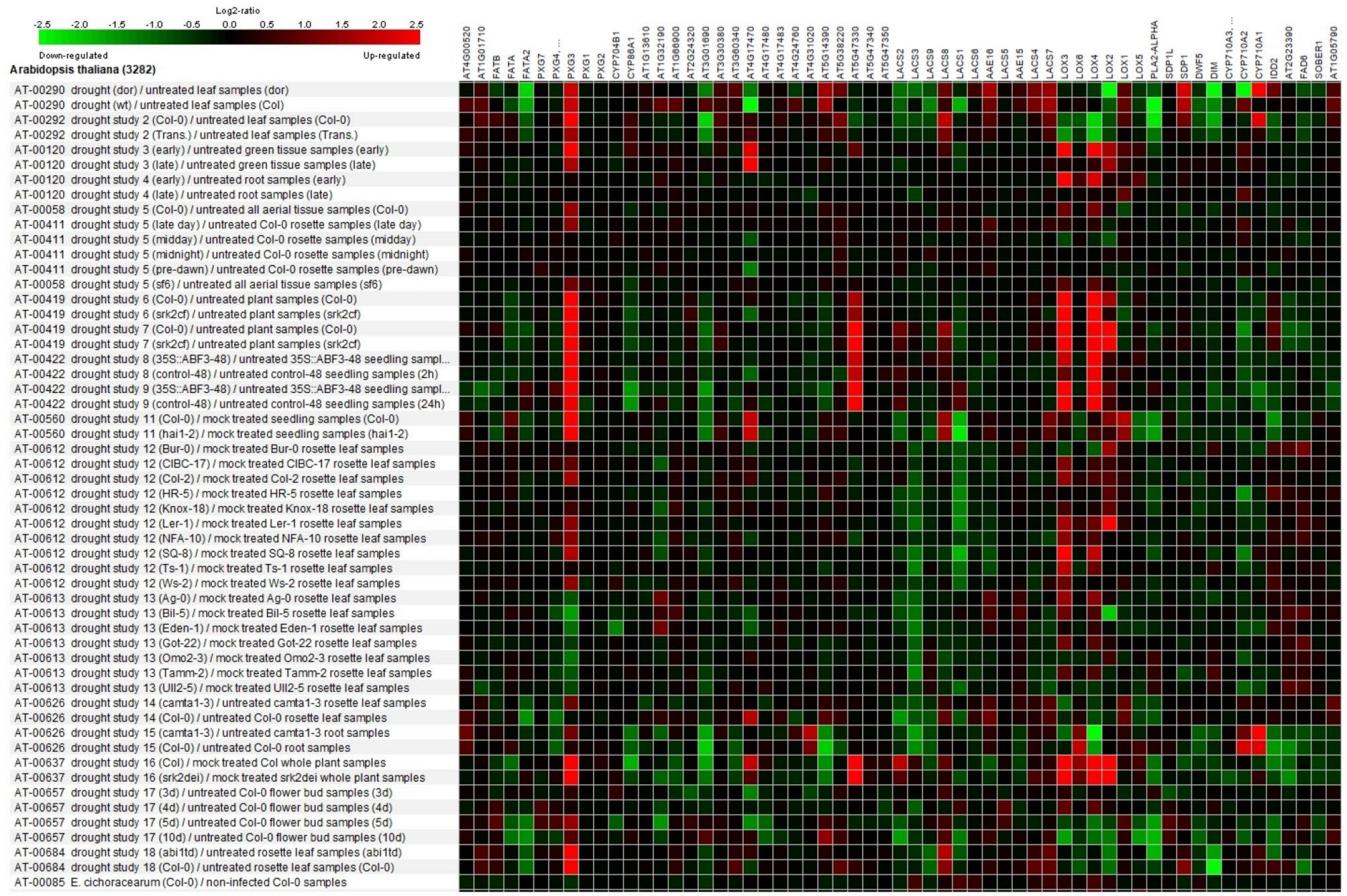
Expression analysis of milk thistle lipids synthesizing genes by Genevestigator software and Biclustering tool. Y-axis contains Arabidopsis gene expression dataset and the X-axis contains our input genes. Red color indicates up-regulated and green color indicates down-regulated genes.

Gene expression patterns of lipid synthesizing genes was extracted from Table 3 and then these genes were analyzed by Genevestigator in *Arabidopsis thaliana* and Biclustering plot was drawn under drought stress (Fig. 3). *PXG3* (*RD20*), *AT4G17470*, *AT5G47330*, *LOX3*, *LOX4*, *LOX2*, *CYP710A1* were the important up-regulated genes, while *FATA2*, *CYP86A1*, *AT3G01690*, *LACS3*, *LACS1*, *PLA2-ALPHA*, and *CYP710A2* down-regulated under drought stress.

### Relationship between gene expression and metabolite variation

Lipids variations and respective involved genes at three different levels of irrigation (Field Capacity (FC), 70% FC, 40% FC) was studied (Table 4). In this table, gene expression was extracted from Fig. 3, and lipid content was measured by GC–MS, mentioned above.

**Table 4:**
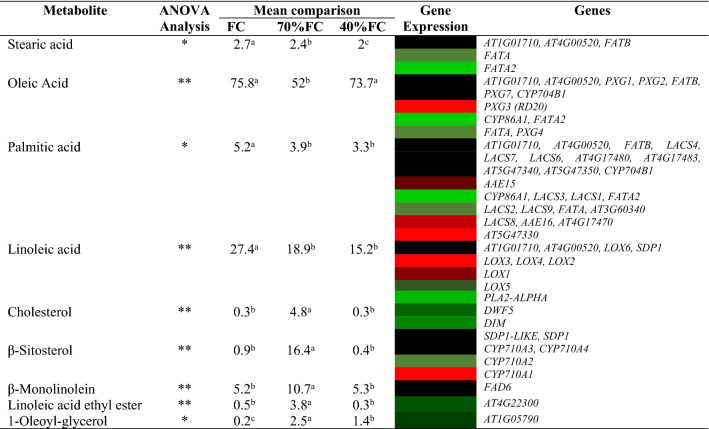
Comparison of lipids variations of milk thistle under three different drought stress levels with their respective biosynthesizing genes expression.

Red color indicates up-regulated and green color indicates down-regulated genes and lipids.

** and * indicate significance at the probability level of 0.01 and 0.05, respectively.

Different letters on top of each number indicate a statistically significant difference between lipid contents of Milk thistle under different drought stress levels.

ANOVA analysis of milk thistle lipids (oily and methanolic) was performed at three different levels of drought stress with three replications. According to the results of Table 4, there was a significant difference between different levels of drought stress in oleic acid, linoleic acid, cholesterol, β-Sitosterol, β-Monolinolein and Linoleic acid ethyl ester at the level of 0.01, but in stearic acid, palmitic acid and 1-Oleoyl-glycerol significant difference at the level of 0.05 was observed.

The stearic acid content decreased with increasing stress intensity. Among genes involved in biosynthesis pathway of stearic acid, a significant decrease and a slight reduction was observed in expression levels of *FATA2* and *FATA*, respectively.

The oleic acid content in treatments FC and 40% FC was extremely high (75.8 and 73.7, respectively), but in treatment 70% FC (52% Area) was lower than two mentioned treatments. In oleic acid biosynthesis pathway, expression of *PXG3* (*RD20*) significantly increased in all pathways, whereas *CYP86A1* and *FATA2* expression significantly reduced.

The amount of palmitic acid has decreased over time with increasing water deficiency. In palmitic acid, expression of *AT5G47330* significantly raised when a drastic decrease of *CYP86A1*, *LACS3*, *LACS1* expression were observed.

The linoleic acid content also decreased under drought stress. Up-regulated genes in linoleic acid biosynthesis pathway were *LOX3*, *LOX4*, *LOX2* and *LOX1* and down-regulated genes were *PLA2-ALPHA* and *LOX5*.

The content of cholesterol and β-Sitosterol in treatment 70% FC had a relatively high value (4.8 and 16.4% Area, respectively), but in two treatments 40% FC and FC was no significant. In cholesterol biosynthesis pathway, expression of *DWF5* and *DIM* decreased. Expression of *CYP710A1* and *CYP710A2* significantly increased and declined, respectively, in β-Sitosterol biosynthesis pathway.

The content of β-Monolinolein was relatively high (10.7% Area) in treatment 70% FC but in the other two treatments the amount was halved. The genes biosynthesizing this lipid did not show significant expression.

The content of Linoleic acid ethyl ester and 1-Oleoyl-glycerol in treatment 70% FC had higher values (3.8 and 2.5% Area, respectively) than the two treatments 40% FC and FC. Expression of linoleic acid ethyl ester and 1-oleoyl-glycerol biosynthesis pathway genes reduced (Table4).

### Relationship between genes and lipids networks

**Figure 4:**
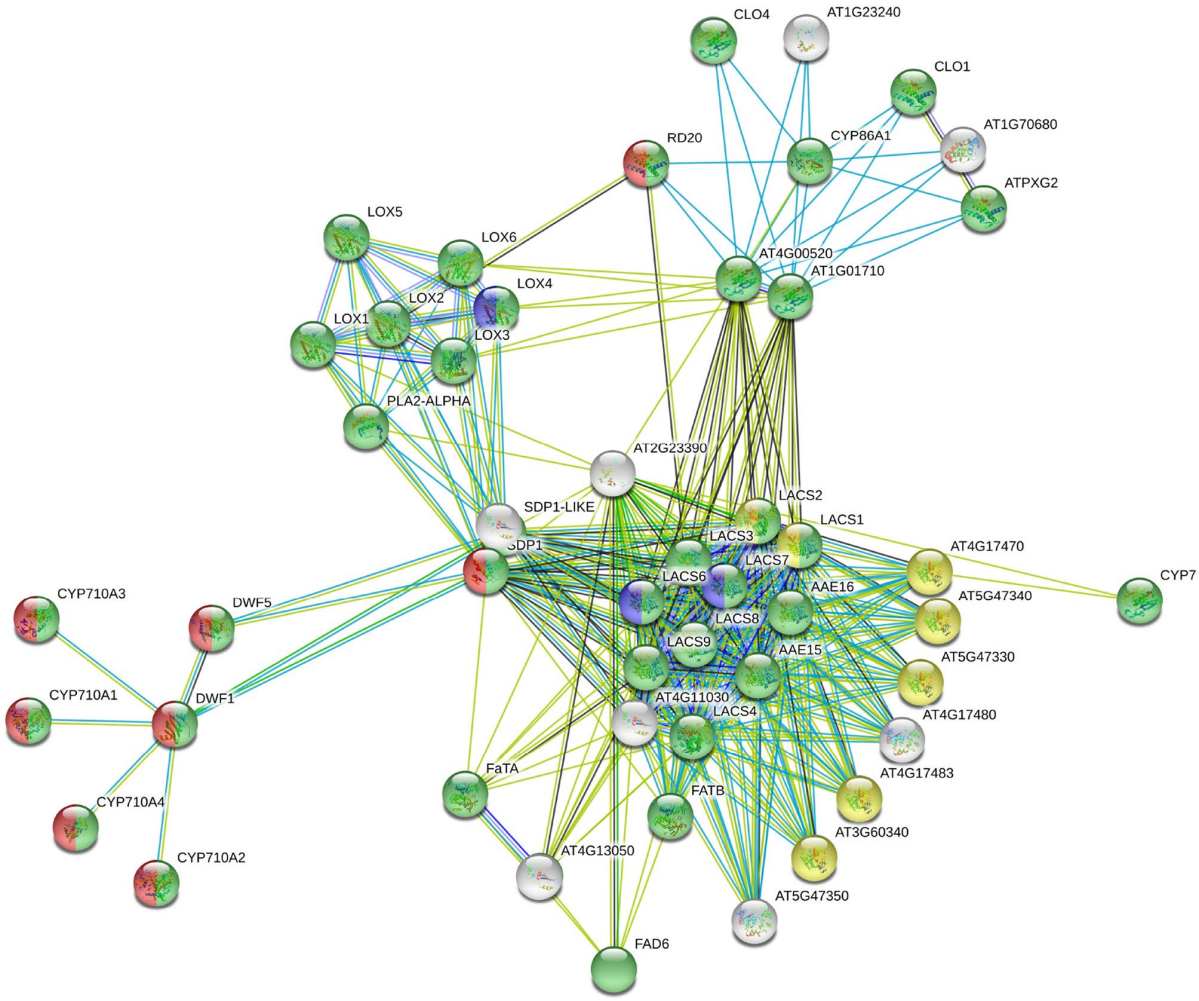
Interaction networks of milk thistle genes. Genes (nodes) with same color activate in the same pathway. Lines between genes indicate the interaction between them.

**Figure 5:**
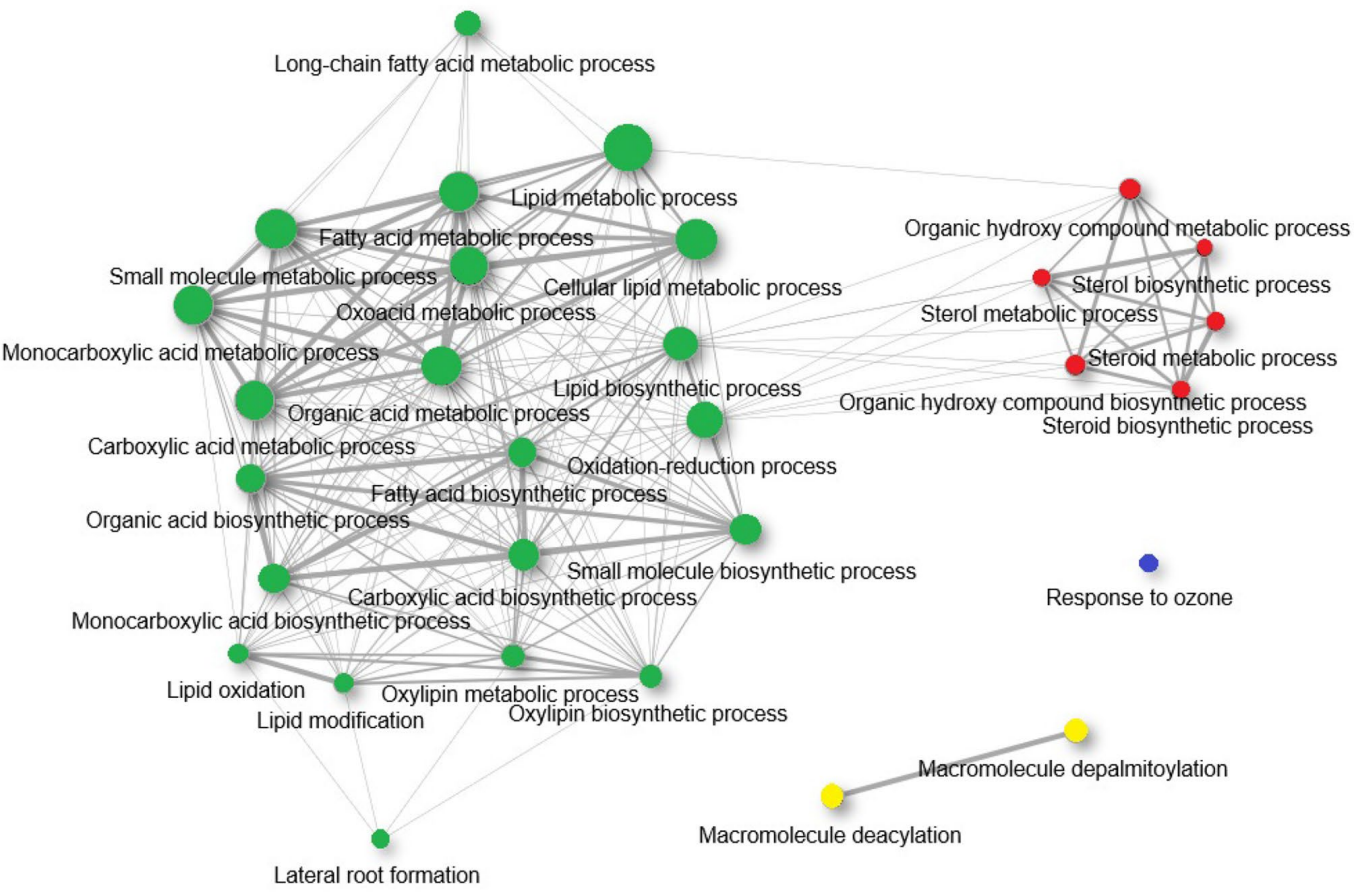
Interaction networks of milk thistle lipids pathways. Each node corresponds to a lipid pathway and the lines between them represent the interaction between them. The identical colors of the nodes indicate the proximity of the respective pathways. These colors correlate with the color of the nodes in the gene network, so that the genes of each color play a role in the lipid pathways of the same color.

For genes and lipids, a network was drawn separately to examine the relationship between genes and lipids. Gene network (Fig. 4) was divided into four groups according to lipid network classification (Fig. 5). There was a correlation between genes and the lipids network, and so, lipid pathways interacted with their biosynthesis genes. The biggest group with maximum number of genes, group 1 (green), included *LOX*, *LACS*, *AAE*, *FAT*, *CYP*, *DWF* and *CLO* families, as well as *FAD6*, *SDP1*, *ATPX62*, *RD20*, *PLA-ALPHA*, *AT1G05790*, *AT4G00520* and *AT1G01710* genes, which led to the biosynthesis of stearic acid, oleic acid, palmitic acid, linoleic acid, cholesterol, β-Sitosterol, β-Monolinolein, linoleic acid ethyl ester and 1-oleyl glycerol. This group of genes is involved in the biosynthetic and metabolic processes of fatty acids, lipids, cellular lipids, small molecules, oxoacid, oxylipin, organic acids, carboxylic acid, monocarboxylic acid, as well as reductions in oxidation, oxidation and modification of lipid, finally, it is involved in the metabolic process of long-chain fatty acid and lateral root formation. Group 2 with red colors comprised *CYP710A1*, *CYP710A2*, *CYP710A3*, *CYP710A4*, *DWF1*, *DWF5*, *SDP1* and *RD20*, which were involved in biosynthesis of cholesterol and β-Sitosterol and organic compounds. Depalmitoylation and deacylation of macromolecules is performed by genes in group3 (yellow), which include *LACS1*, *LACS2*, *AT4G17470*, *AT5G47340*, *AT5G47330*, *AT4G17483* and *AT3G60340* were categorized into group 3, yellow colors, which were active in palmitic acid and linoleic acid ethyl ester biosynthesis pathway. Group 4 with blue colors contained *LACS6*, *LACS7* and *LOX4*, which engaged in biosynthesis pathway of palmitic acid and linoleic acid play role in the response to ozone.

After comparing protein and metabolite networks, 4 up- and 4 down-regulated genes with high interaction was chosen (Fig. 4). Given that *CYP86A1*, *CYP710A1*, *FATA2*, *LACS3*, *LOX2*, Palmitoyl-protein thioesterase (*PAL*), *PLA2-ALPHA* and *PXG3* genes were not previously identified in the plant of our interest, so, the nucleotide sequence of these genes (based on our RNA-Seq data in previous research) was provided to submit in NCBI database. Accession numbers of selected genes are MW151571, MW151572, MW151573, MW151574, MW151575, MW151576, MW151577 and MW151578, respectively.

### Gene expression analysis using qRT-PCR

To confirm the expression of selected genes in milk thistle, gene expression analysis was performed for 4 up-regulated and 4 down-regulated genes.

Expression of 4 up-regulated genes, *PXG3*, *LOX2*, *CYP710A1* and Palmitoyl-protein thioesterase (*AT5G47330*), and 4 down-regulated genes, *FATA2*, *CYP86A1*, *LACS3* and *PLA2-ALPHA*, was compared at three irrigation levels (FC, 70% FC and 40% FC) with three replications.

**Figure 6:**
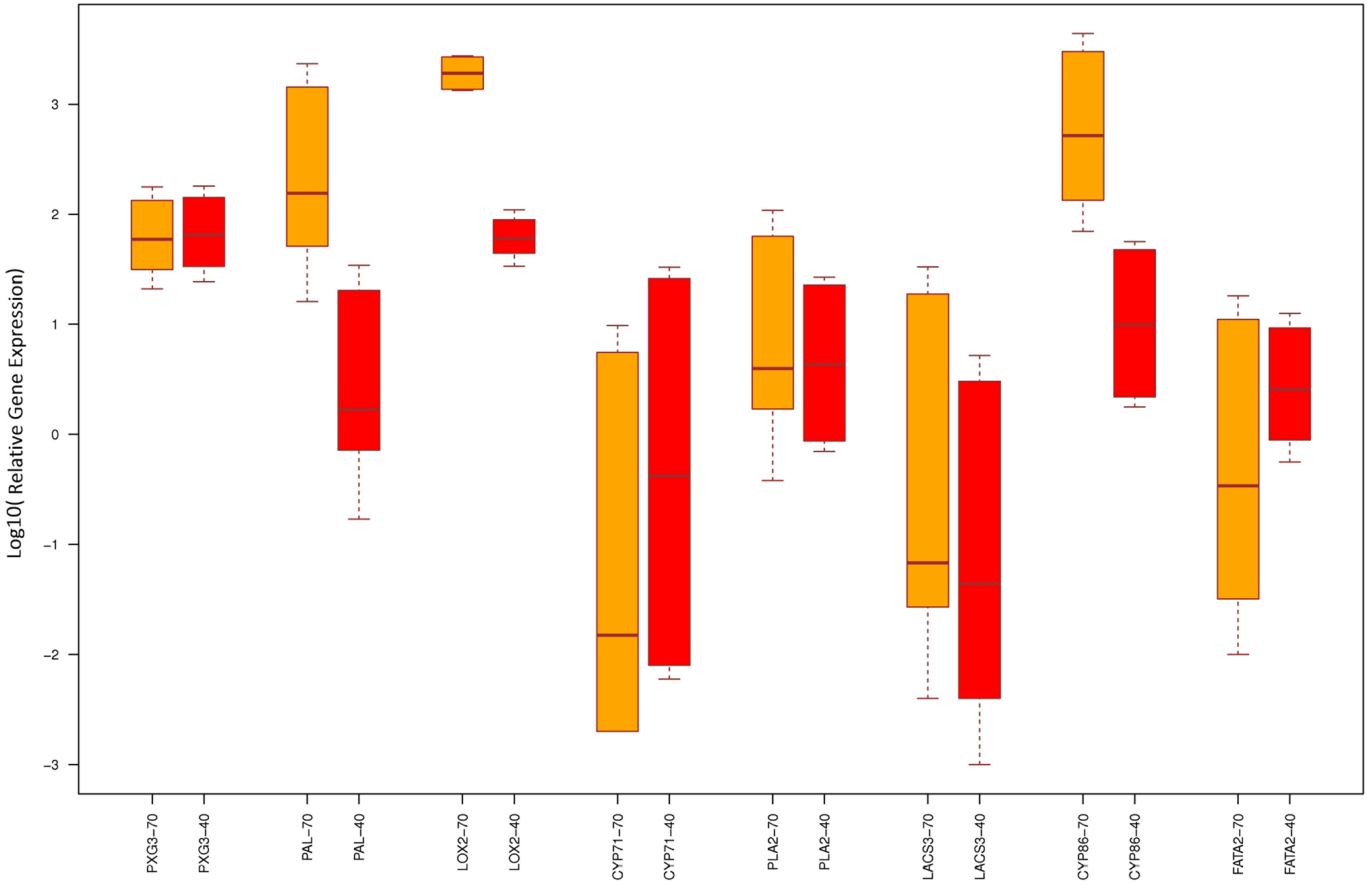
Relative expression analysis of 8 hub genes (4 up-regulated and 4 down-regulated genes) at 70%FC (orange color) and 40% FC (red color) versus FC treatment (field capacity). The relative expression of genes is shown based on Log10. Because the differences in expression between the different genes were so large, logarithms were used to make them easier to display and compare better.

*PXG3* gene expression increased 65.119 and 59.302 times in 40% FC and 70% FC treatments compared to FC, respectively. The expression of this gene was relatively similar in both treatments and had a significant increase compared to the control. Palmitoyl-protein thioesterase (*PAL*) expression significantly increased compared to control in treatment 70% FC (154.879) but no significant increase of expression was observed in treatment 40% FC (1.693). Expression of *LOX2* was significantly increased in both 40% FC and 70% FC treatments while expression level of this gene in treatment 70% FC (1917.49) was significantly higher than treatment 40% FC (60.129) compared to control. A reduction of *CYP86A1* gene expression was observed in both 40% FC and 70% FC treatments compared to the control (0.419 and 0.015, respectively). The expression of *PLA2-ALPHA* gene in two treatments 40% FC and 70% FC increased by 4.332 and 3.972, respectively, compared to the control although this increase was not significant. Expression of *LACS3* was reduced in the 40% FC and 70% FC treatments at ratio of 0.044 and 0.068, respectively, compared to the control. *CYP86A1* expression in both treatments 40% FC and 70% FC showed a significant increase compared to the control. Increased expression of this gene in treatment 70% FC (519.147) was significantly higher than treatment 40% FC (9.952) compared to control. *FATA2* gene in treatment 40% FC showed an increase compared to FC (2.585), which was not significant while the expression of this gene decreased in treatment 70% FC (0.343) (Fig. 6).

### Protein homology modeling and reliability of modeled structures

**Figure 7:**
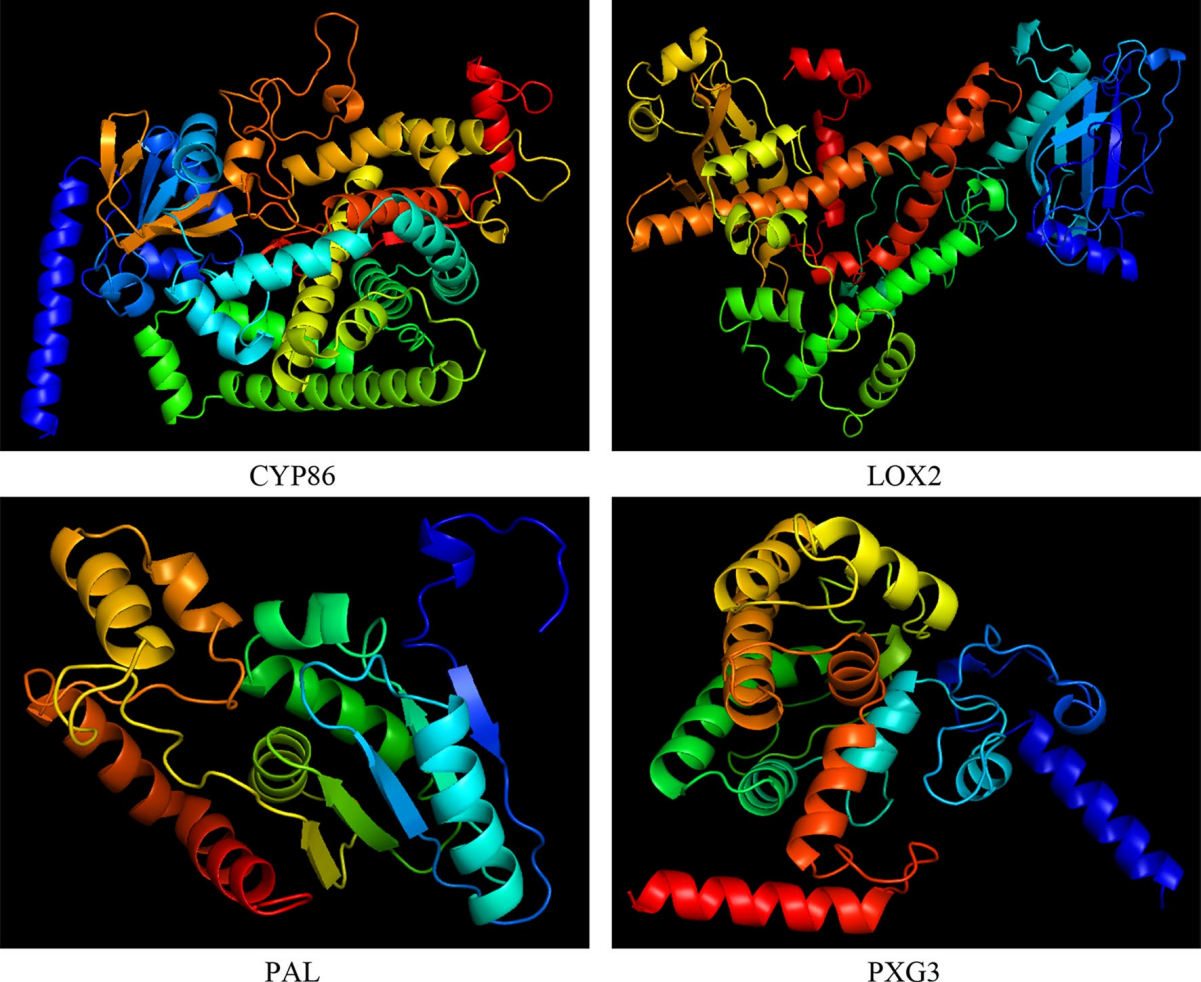
Modeled structures of the selected four proteins with cartoon representation in milk thistle. Proteins are colored by spectrum from N to C terminus. The confidence (TM-score) of the predicted models in CYP86, LOX2, PAL and PXG3 are high (0.544), low (0.325), high (0.518) and medium (0.467), respectively.

Four selected proteins (CYP86, LOX2, PAL and PXG3) with high expression level were selected and their structure were modeled. The model with the highest Confidence and TM-score was chosen for each protein among suggested five models by TrRosetta server (Fig. 7). Homology modeling information was acquired for each protein using trRosetta server and presented in Table 5. CYP86 and PAL had a high Confidence and a TM-score (0.544 and 0.518, respectively). PXG3 showed a medium confidence and a TM-score of 0.467, while LOX2 had low confidence and TM-score (0.325).

**Table 5:** Features of the predicted protein models on two servers, trRosetta and ProFunc.

trRosetta server			ProFunc server			
Protein	Confidence	TM-score	Most favoured regions (%)	Additional allowed regions (%)	Generously allowed regions (%)	Disallowed regions (%)
CYP86	High	0.544	91.2	7.3	1.3	0.2
LOX2	Low	0.325	87.8	11.6	0.2	0.4
PAL	High	0.518	85.3	13.6	1.1	0.0
PXG3	Medium	0.467	88.1	11.1	0.9	0.0

trRosetta server includes confidence and TM-score, and ProFunc server includes most favoured regions (%), additional allowed regions (%), generously allowed regions (%) and disallowed regions (%).

TM-score is between 0 and 1 and a TM-score higher than 0.5 usually indicates a model with correctly predicted topology^41^.

**Figure 8:**
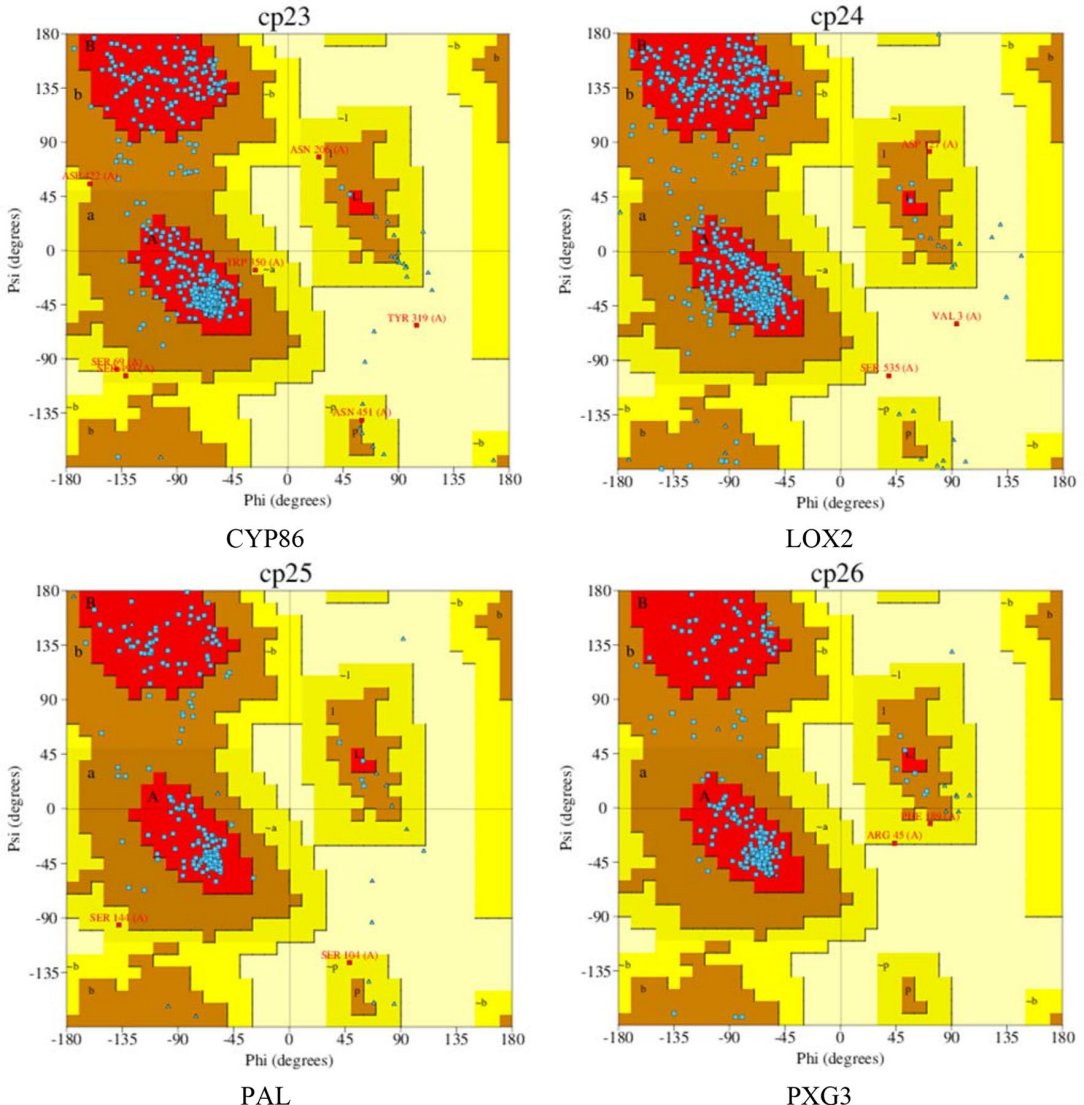
Ramachandran plots of the selected proteins. Most favoured regions in the Ramachandran plots are shown in red; additional allowed regions are shown in brown; generously allowed regions are shown in yellow and disallowed regions are shown in light yellow color. Blue color dots represent (φ, ψ) angles for each residue of the predicted structure.

Application of Ramachandran plot with ProFunc server showed that 91.2, 87.7, 85.3 and 88.1% of amino acids in CYP86, LOX2, PAL and PXG3, in an order, were in the most favored regions, where the maximum points are observed (Fig. 8). Percentage of amino acid dispersion in additional allowed regions, generously allowed regions and disallowed regions are listed in Table 5.

### Function analysis of selected proteins

Protein analysis using CATH database exhibited cholesterol 24-hydroxylase isoform X2 function for CYP86, Lipoxygenase function for LOX2, palmitoyl-protein thioesterase function for PAL, and, probable calcium-binding peroxygenase function for PXG3.

### Molecular docking

**Figure 9:**
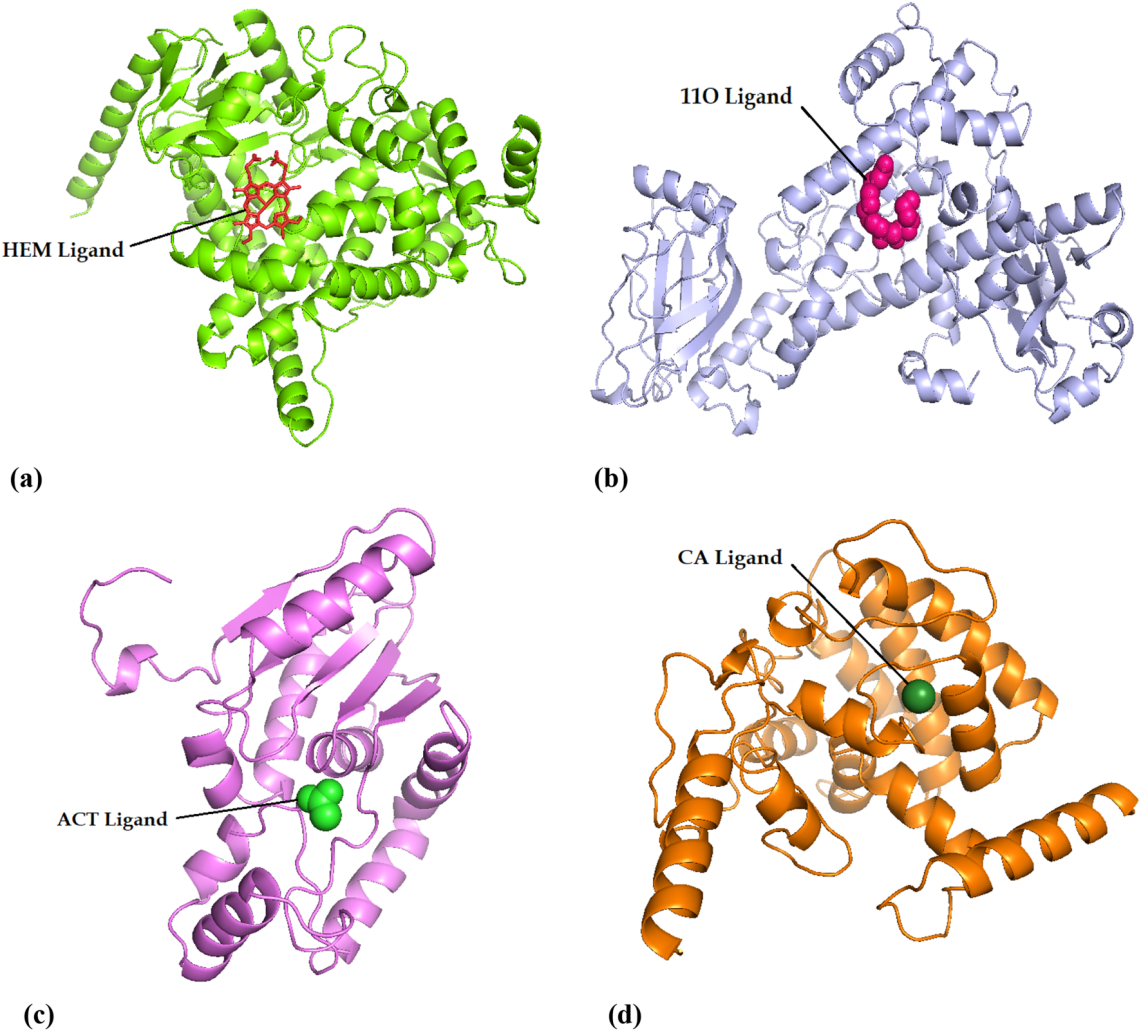
Cartoon representation of selected proteins interaction with their ligands. (**a**) CYP86 protein with HEM ligand. (**b**) LOX2 protein with 11O ligand. (**c**) PAL protein with ACT ligand. (**d**) PXG3 protein with CA ligand.

The most plausible predicted ligands for CYP86, LOX2, PAL and PXG3 proteins were HEM, 11O, ACT and LIG, respectively (Fig. 9). The binding energies and C-scores are listed in Table 6.

**Table 6:**
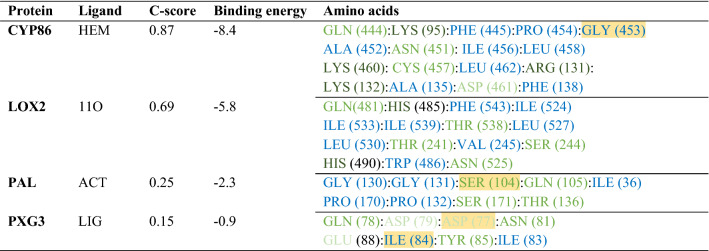
Ligand and protein interacting amino acids in 5A and their characteristics.

Polar amino acids: green, non-polar amino acids: blue, positively charged amino acids: dark green, negatively charged amino acid: light green, hydrogen bond: yellow highlight.

**Figure 10:**
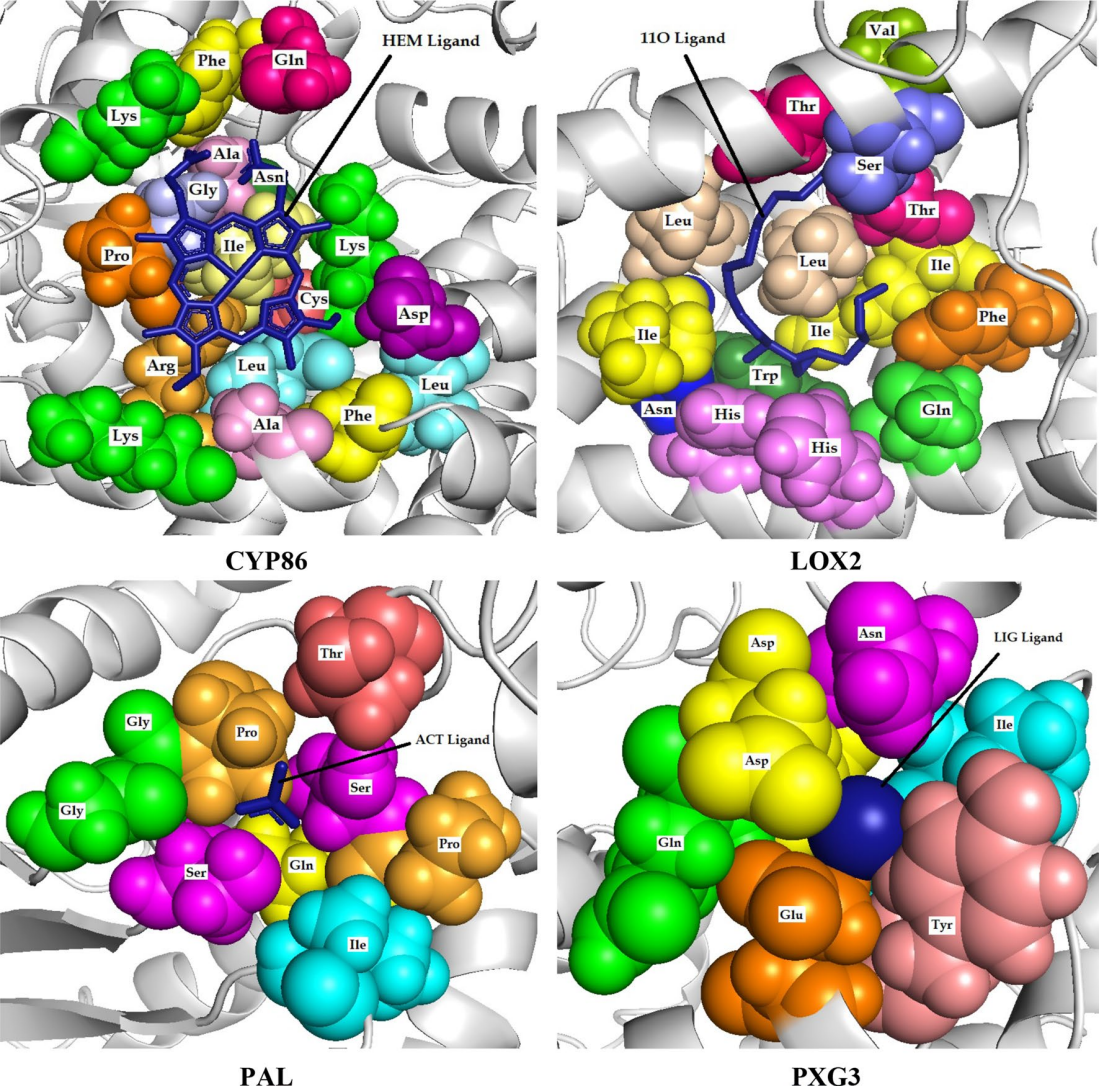
Interactions between the ligand and amino acids within 5A. Amino acids are shown as spheres and the ligands as cartoon representations. For better detection, each amino acid is shown with a specific color.

HEM ligand interacted with 17 amino acids of CYP86 protein, listed in Table 6 and Fig. 10. Analysis of the atomistic interactions revealed that eight polar (GLN:444, LYS:95, ASN:451, LYS:460, CYS:457, ARG:131, LYS:132, ASP:461) and nine non-polar (PHE:445, PRO:454, GLY:453, ALA:452, ILE:456, LEU:458, LEU:462, ALA:135, PHE:138) interactions were formed between HEM and CYP86. The ligand also formed a hydrogen bond with amino acid GLY: 453. 11O ligand in LOX2 interacted with 15, 7 polar (GLN:481, HIS:485, THR:538, THR:241, SER:244, HIS:490, ASN:525) and eight non-polar (PHE:543, ILE:524, ILE:533, ILE:539, LEU:527, LEU:530, VAL:245, TRP:486), amino acids. In addition, no hydrogen bond was observed between ligand and amino acids. In PAL, ACT ligand had interaction nine amino acids, four polar (SER:104, GLN:105, SER:171, THR:136) and five non-polar (GLY:130, GLY:131, ILE:36, PRO:170, PRO:132) amino acids. ACT formed a hydrogen bond with amino acid SER:104. Ligand LIG in PXG3 interacted with eight amino acids, six polar (GLN:78, ASP:79, ASP:77, ASN:81, GLU:88, TYR:85) and two non-polar (ILE:84, ILE:83). LIG formed a hydrogen bond with two ASP and ILE amino acids.

## Discussion

Lipids as vital and major cellular constituents provide a structural basis for cell membranes and an energy resource for metabolism. In addition, lipids as signal mediators are involved in initiation of defense reactions^42^, as well as mitigation processes in response to stress in plant cells^43^. Therefore, lipid contents including fatty acids, hydrocarbons, esters, steroids, and etc. are affected by different stress conditions. For example, dehydration drastically reduced and altered lipid levels and compositions^44^. Thus, finding genes biosynthesizing these lipids helps us understand the mechanism of stress tolerance. Among genes involved in lipids synthesis, differentially expressed genes under drought stress were chosen. These genes included four up-regulated *CYP710A1*, *LOX2*, *PXG3*, and Palmitoyl-protein thioesterase (*PAL*), and four down-regulated, *FATA2*, *CYP86A1*, *LACS3* and *PLA2-ALPHA* genes.

*CYP710A1* and *CYP86A1*, as members of cytochrome P450 gene family, protect plants against multiple biotic and abiotic stresses through involving in plenty of detoxification activities and biosynthetic pathways^45^.

*LOX*s act in signaling processes due to stressor effects and lipoxygenase activity characteristics, and may serve as molecular markers for plant stress tolerance studies^46^. Probable calcium-binding peroxygenases (*PXG3*) take part in storage lipid degradation of oil bodies in the abiotic stress signaling pathways as well as in drought tolerance by controlling stomata under water deficiency^47^. Palmitoyl-protein thioesterase (*PAL*) is responsible for removing palmitate group from its substrate proteins, which might contain cysteine string protein (*CSP*), presynaptic proteins like SNAP-25, dynamin, and synaptotagmin^48^.

*FATA2* plays an important function in chain termination within de novo synthesis of fatty acid and is also essential for plant viability^49^. Long-chain acyl-CoA synthetases (*LACS*s) synthesize long-chain acyl-CoAs from free fatty acids in plant cells. *LACS2* is primarily involved in polyunsaturated linolenoyl-CoA production, which is vital to activate ethylene response transcription factors-mediated hypoxia signaling^50^. *PLA2*, as one of phospholipase A2, manages plenty of cellular processes, such as development, growth, defense, and stress responses^51^.

The results of gene expression in milk thistle were similar to the results of the Genevestigator program in Arabidopsis under different drought experiments. Under drought stress conditions, expression of four genes *CYP86A1*, *LOX2*, Palmitoyl-protein thioesterase (*PAL*) and *PXG3* increased while the other four genes did not change significantly.

To further investigate function mechanism of these genes, protein structure of the respected genes was examined. Due to lack of protein structure sequenced in milk thistle, both quality of homology modeling and accuracy to dock substrate to the substrate-binding site^52^ were taken into account. Confidence of CYP86A1 and PAL was high, while for PXG3 and LOX2 were medium and low, respectively. The constructed models examined by Ramachandran analysis exhibited that these models are relatively close to reality. Protein interaction with the respective ligands was investigated to analyze their atomic interactions and functional preferences.

Although approximately 1% of plant protein-coding genes are predicted to encode P450s^53^, just seven P450 protein crystals of plants were found in Protein Data Bank (PDB) database because it is localized in the membrane and the structure is simply degraded and broken within purification and crystal growth^54^. Heme-containing P450s bonded to oxidoreductases catalyze stereo- and regio-selective oxidations, and also intensively challenging reactions, including decarboxylation, deamination, C–C cleavage, ring opening, coupling, expansion, migration, and dehydration^55^.

For understanding the architecture of HEM binding site, one of biologically active motifs present in CYP, is crucial to establish the mechanism assembling basic metabolic machinery and facilitating secondary metabolites formation. It was previously reported that Phenylalanine, Glycine, Alanine, Arginine, Isoleucine, Cysteine and Proline amino acids were frequently repeated in HEM binding site of nine investigated plants by a proteome-wide identification of CYP enzymes analysis using statistical weight matrix approach^56^. Our results confirmed that the mentioned amino acids interacted with the HEM ligand. Residues interacting with HEM are mostly non-polar, particulalry aromatic amino acids, preparing a hydrophobic condition for HEM ring structure^57^. Our results demonstrated that nine amino acids interacting with HEM ligand were non-polar, which one of them had a hydrogen bond. Hydrogen bonds play important roles in protein folding^58^, protein–ligand interactions^59^, and catalysis^60^ and affect molecule physicochemical properties, such as distribution, partitioning, solubility, and permeability, which are essential to drug development^60^. A mutation in conserved glycine of heme-binding motif led to an inactive and unstable apoprotein, which supports a main function of glycine to bind the heme and probably regulating P450s activity^61^. Therefore, glycine mutant instability extremely restricts examination of its exact catalytic role^62^.

LOX2, a member of a large monomeric protein family with non-sulphur, non-heme, and iron cofactor containing dioxygenases, interacted with ligand 11O. Free fatty acids are considered to be the main substrates of LOX although phospholipids and glycerolipids were also identified as oxygenation substrates^63^. Lipoxygenases (LOXs) catalyze polyunsaturated fatty acid (PUFA), as a substrate, to synthesize hydroperoxides^64^. Therefore, iso-enzymes of LOX would be identified according to substrate’s peroxidation site^65^. LOXs generate HPOs, which act as reactions hubs because of having cytotoxic potential to cell membrane, therefore, are rapidly metabolized in chemical compounds engaged in plant defense, signaling and apoptosis^66^. Since the majority of studies have focused on compounds with lipoxygenases inhibitory activity, investigation of ligand 11O and its binding site has not undertaken yet and should it be taken into account for further studies.

PXG3, a member of caleosin family, interacted with CA ligand. PXG3 protein binding site includes two hydrogen bonds between two ASP and ILE amino acids with calcium ligand. Caleosins have a specific calcium-binding domain in N-terminal region^67^, a hydrophobic domain and a proline knot motif probably to target protein to oil bodies (OBs)^68^. In Arabidopsis, Peroxygenase activity of AtCLO1 and AtCLO2, two members of caleosin family, required the presence of calcium and two conserved histidines, which subsequently proved by Aubert et al.^69^ that these two histidines were in the PXG3 sequence. Despite acting PXG3 as the putative OB-associated peroxygenase, it may participate in lipid modifications or signaling in stress responses of plants^69^. Biosynthesis of cuticular waxes and cuticle was increased under water and ABA deficiency and in Arabidopsis^70^, as a result of peroxygenase activity in wax and cuticle synthesis pathway^71^. It was exhibited that PXG3 takes part in drought tolerance mechanisms by regulating plant growth, stomatal aperture and water use efficiency^69^.

Palmitoyl-protein thioesterase interacted with ACT ligand. This protein was known only in Arabidopsis and no attributed function has been reported yet.

Lipid composition indicated a main impact on cellular membrane integrity and activities of intrinsic-membrane proteins under stress conditions^72^. Therefore, preservation of cellular membrane integrity, as a key factor to retain metabolic homeostasis^73^, is a prerequisite to survive during severe stress conditions^74^. In addition to identifying important lipids and genes involved in their synthesis, the method of identifying lipids is also of particular importance. Characterization and identification of main compounds in biological processes, such as metabolites, lipids and proteins have been facilitated by implementing high sensitivity and resolution in mass spectrometry (MS)^75^, highlighting modern lipidomic tools can be applied to study of plant lipid^76^ by relying on tailored and optimized MS-based strategies^77^.

## Conclusion

Our results highlighted the importance of lipids as main components of plant cells, which play a major role in stress conditions. Regulating gene expression is one of mechanisms in plants to reduce the stress influences. Lipid components and respected biosynthesizing genes of milk thistle were identified. Determined the genes of these lipids and examined their expression in various plants under drought stress. Next, we selected 8 genes (*PXG3*, *LOX2*, *CYP710A1*, *PAL*, *FATA2*, *CYP86A1*, *LACS3*, and *PLA2-ALPHA*) that had differential expression and examined their expression in milk thistle. Out of 8 selected genes, 4 that had high expression (*PXG3*, *PAL*, *LOX2*, and *CYP86A1*) were selected, and protein structures were obtained by homology modeling. Next, the ligands attached to these proteins were examined. Examination of ligands, binding site amino acids, and atomistic interactions confirmed that the interactions between proteins and ligands are powerful and useful. The product of these proteins, which contains lipids, also plays the final role in confirming the role of these genes in stress tolerance.

## Data Availability

The sequence of genes studied in this paper includes CYP86A1, CYP710A1, FATA2, LACS3, LOX2, Palmitoyl-protein thioesterase (PAL), PLA2-ALPHA and PXG3 are available on the National Center for Biotechnology Information (NCBI) Nucleotide database with the Accession number of MW151571, MW151572, MW151573, MW151574, MW151575, MW151576, MW151577 and MW151578, respectively.
